# The Proportion of Uncoded Diagnoses in Computerized Health Insurance Claims in Japan in May 2010 According to ICD-10 Disease Categories

**DOI:** 10.2188/jea.JE20130194

**Published:** 2014-09-05

**Authors:** Shinichi Tanihara

**Affiliations:** Department of Public Health and Preventive Medicine, School of Medicine, Fukuoka, Japan; 福岡大学医学部衛生公衆衛生学

**Keywords:** health insurance claim, uncoded diagnoses, ICD-10

## Abstract

**Background:**

Uncoded diagnoses in computerized health insurance claims are excluded from statistical summaries of health-related risks and other factors. The effects of these uncoded diagnoses, coded according to ICD-10 disease categories, have not been investigated to date in Japan.

**Methods:**

I obtained all computerized health insurance claims (outpatient medical care, inpatient medical care, and diagnosis procedure-combination per-diem payment system [DPC/PDPS] claims) submitted to the National Health Insurance Organization of Kumamoto Prefecture in May 2010. These were classified according to the disease categories of the International Statistical Classification of Diseases and Related Health Problems, 10th Revision (ICD-10). I used accompanying text documentation related to the uncoded diagnoses to classify these diagnoses. Using these classifications, I calculated the proportion of uncoded diagnoses by ICD-10 category.

**Results:**

The number of analyzed diagnoses was 3 804 246, with uncoded diagnoses accounting for 9.6% of the total. The proportion of uncoded diagnoses in claims for outpatient medical care, inpatient medical care, and DPC/PDPS were 9.3%, 10.9%, and 14.2%, respectively. Among the diagnoses, *Congenital malformations, deformations, and chromosomal abnormalities* had the highest proportion of uncoded diagnoses (19.3%), and *Diseases of the respiratory system* had the lowest proportion of uncoded diagnoses (4.7%).

**Conclusions:**

The proportion of uncoded diagnoses differed by the type of health insurance claim and disease category. These findings indicate that Japanese health statistics computed using computerized health insurance claims might be biased by the exclusion of uncoded diagnoses.

## Abstract (ja)

**【はじめに】:**

診療報酬明細書（レセプト）の電子化が進展したが未コード化傷病名はレセプトを用いた政府統計や調査における活用が困難である。未コード化傷病名の存在がレセプト分析に与える影響を検証する目的で国際疾病分類第10版（ICD10）の大分類別に未コード化傷病名割合を検討した。

**【方法】:**

熊本県国民健康保険団体連合会にオンラインもしくは電子媒体で提出された2010年5月診療分のレセプト（医科入院外、医科入院、DPC/PDPS）に記載された全ての傷病名をICD10対応標準病名マスターに沿ったコード化傷病名と未コード化傷病名に分類した。コード化傷病名はICD10の大分類に沿って再分類した。未コード化傷病名は当該傷病名に対応してテキスト入力された傷病名を用いて分類した。医科入院外、医科入院、ＤＰＣの種類別に大分類別の未コード化傷病名の割合を集計した。

**【結果】:**

分析に用いた傷病名総数3,804,246件の内363,753件 (9.6%)が未コード化傷病名であった。医科入院外、医科入院、DPC/PDPSの未コード化傷病名割合はそれぞれ9.3%、 10.9%、 14.2%とレセプトの種類によって未コード化傷病名割合が異なっていた。大分類別では最大値「先天奇形、変形及び染色体異常」（大分類20）の19.3%と最小値「呼吸器系の疾患」（大分類10）の4.7%に４倍以上の格差を認めた。

**【結論】:**

未コード化傷病名の出現頻度はレセプトの種類や傷病大分類によって異なっていた。電子化されたレセプトを分析する上で未コード化傷病名の存在は無視できないと考えられた。

## INTRODUCTION

A health insurance claim (HIC) is a document prepared by healthcare providers for reimbursement of the cost of healthcare services. Usually, an HIC record contains: (a) the patients’ sex and date of birth, (b) health insurance qualification status, (c) procedures and drugs provided, and (d) diagnoses according to the condition of the patient. The information described in HICs contained in large administrative databases has been used to evaluate drug safety^1^ and estimate the prevalence of specific diseases^2^ and facilitate surveillance for surgical site infections,^3^ as well as to assess the incidence of idiopathic nephritic syndrome factors that predispose patients to develop end-stage renal disease,^4^ the incidence of adverse events of medical procedures,^5^ the association between cardiovascular disease and the risk of major osteoporotic fracture,^6^ and the cause of death among patients with amyotrophic lateral sclerosis.^7^ In Japan, the completeness of the infectious disease surveillance system,^8^ quality of care for diabetes patients,^9^ and relationships between health guidance for metabolic syndrome and outpatient charges or drug costs related to metabolic syndrome^10^ have been evaluated using the information described in HICs. However, there are some limitations to using large administrative databases of HICs in Japan. For example, the linkage rate between the results of health check-ups and HICs has been reported to be very low.^11^ The reliability of administrative databases using HICs in Japan should be investigated more precisely.

Due to the regulations surrounding medical cost reimbursement in Japan, health care providers are required to submit a single HIC describing all of the health care services rendered by the provider for an individual in a given calendar month. This means that if a patient visits one health care provider more than once in a given calendar month for two different diseases, such as for hypertension in the beginning of the month and for acute bronchitis at the end of the month, the health care provider submits only one HIC. Therefore, most HICs contain more than one diagnosis.^12^^,^^13^ In the past, most HICs were submitted on paper, which caused technical limitations affecting the handling of the information in the HICs; it has been common for only one principal diagnosis to be selected from an HIC when estimating disease-specific medical expenditures.^14^ Among the elderly insured by the National Health Insurance for Medical Services for the Aged, there has been a tendency to select hypertension as the principal diagnosis, which has led to overestimates of disease-specific medical expenditures.^12^

After August 2010, all hospitals and medical clinics were required to submit electronic HICs to claim reimbursement for the costs of health care services. Subsequently, 93.1% of HICs were computerized by March 2011.^15^ The computerized HICs in Japan contain diagnosis codes based on the International Statistical Classification of Diseases and Related Health Problems, 10th Revision (ICD-10). If the medical facilities were unable to code a diagnosis, they were classified as “uncoded” and the medical facilities submitted HICs with text documentation related to these uncoded diagnoses. The uncoded diagnoses are not used in creating summaries of key health statistics.

It has been reported that the proportion of uncoded diagnoses was 9.5% in Japan in December 2010.^16^ However, the causes of uncoded diagnoses remain unclear. The main problem of uncoded diagnoses is that the investigation of large administrative databases using HICs in Japan may have been biased if there was a tendency for one or more specific diagnosis to be uncoded more often than others. For the same reasons, the estimation of disease-specific medical expenditures using HICs may have been biased.^10^^,^^13^ The purpose of this study is to identify the tendencies for specific diagnoses to be left uncoded.

## METHODS

### HICs in this study

In Japan, health insurance coverage is universal and based on fee-for-service reimbursement. Charges for patients vary by the patient’s condition and disease and by the number of procedures and drugs provided. To claim reimbursement for the costs of health care services in a given calendar month, excluding coinsurance, health care providers submit an HIC for each patient treated to Health Insurance Claims Review and Reimbursement Services or to the National Health Insurance Organization (NHIO) located in the same prefecture as the medical facilities used by the patient. The place of submission depends on the patients’ health insurance. The HICs are investigated to determine the patients’ qualification status and if the health care services provided meet the regulations of the reimbursement rules. Reimbursement rules dictate that each clinical procedure must be justified by a corresponding diagnosis. Thus, an HIC contains: (1) the patients’ qualifying information, including name, sex, and date of birth; (2) the procedures and drugs provided; and (3) the diagnoses according to the condition of the patient.

Outpatient and inpatient care are charged separately in Japan, and different HICs are used for these types of care. In 2003, the Diagnosis Procedure Combination/Per-Diem Payment System (DPC/PDPS) was introduced in hospitals certified for acute inpatient care. Thus, there are now three types of HICs in Japan: outpatient care, inpatient care, and DPC/PDPS. For outpatient care, every health provider submits uniform HICs. For inpatient care, hospitals not certified for DPC/PDPS submit inpatient HICs, while hospitals certified for DPC/PDPS submit DPC/PDPS claims for certified acute inpatient care. Even if hospitals are certified for DPC/PDPS, inpatient HICs are submitted for inpatient care that is not part of DPC/PDPS.

Every computerized HIC in Japan contains diagnostic codes based on ICD-10. In addition, every diagnosis is accompanied by supporting text documentation. In this study, all three types of HICs electronically submitted to the NHIO of Kumamoto Prefecture in May 2010 were obtained. The diagnoses were classified according to the disease categories in ICD-10, which are subdivided into chapters. To classify uncoded diagnoses, I used the text documentation accompanying the uncoded diagnoses and calculated the proportion of uncoded diagnoses by ICD-10 chapter.

### Statistical analysis

The proportions of uncoded diagnoses among outpatient care, inpatient care, and DPC/PDPS were compared. Descriptive summary statistics were evaluated as frequencies and proportions for categorical data. The χ^2^ test was used for statistical analyses. A two-sided *P* value of <0.05 was considered statistically significant. All analyses were performed using IBM SPSS Statistics, Version 19 (International Business Machines Corporation, Armonk, NY, USA).

### Ethical concerns

All personal information from HIC data was deleted by the NHIO before the data were given to the researcher. This study was given ethical approval by the Institutional Review Committee of Fukuoka University.

## RESULTS

Table 1 shows the number of analyzed diagnoses. Among the 3 804 246 diagnoses that were included in the data, 3 393 106 (89.2%) were from outpatient medical care HICs, 325 968 (8.6%) were from inpatient medical care HICs, and 85 172 (2.2%) were from DPC/PDPS claims. The number of uncoded diagnoses was 363 753 (9.6%). The proportions of uncoded diagnoses for outpatient medical care, inpatient medical care, and DPC/PDPS were 9.3%, 10.9%, and 14.2%, respectively. The differences were statistically significant (*P* < 0.001).

**Table 1.** Number of diagnoses in HICs and the proportion of non-coded diagnoses according to the type of claim

	The number of diagnoses			

Types of HIC	Uncoded diagnoses	(%)	Total diagnoses	*P*
Outpatient	316 151	9.3%	3 393 106	
Inpatient	35 493	10.9%	325 968	<0.001
DPC/PDPS	12 109	14.2%	85 172	
Total	363 753	9.6%	3 804 246	

DPC/PDPS, Diagnosis Procedure Combination/Per-Diem Payment System; HIC, health insurance claim.

Table 2 shows the proportion of uncoded diagnoses according to the type of HIC and major disease categories. The number of uncoded diagnoses that I was unable to classify according to the disease categories of the ICD-10 using the text documentation was 27 725. *External causes of morbidity and mortality* (Major disease category 20) was not found in outpatient or inpatient HICs. There were 58 125 DPC/PDPS claims in this category and no uncoded diagnoses. Among all diagnoses, *Congenital malformations, deformations, and chromosomal abnormalities* (Major disease category 17) had the overall highest proportion of uncoded diagnoses (19.3%). This category also had the highest percentage of uncoded diagnoses in outpatient medical care HICs (19.5%). For inpatient medical care HICs, *Injury, poisoning, and certain other consequences of external causes* (Major disease category 19) had the highest proportion of uncoded diagnoses (19.7%). For DPC/PDPS, *Neoplasms* (Major disease category 2) had the highest proportion of uncoded diagnoses (77.9%). Among all claims, *Diseases of the respiratory system* (Major disease category 10) had the lowest proportion of uncoded diagnoses (4.7%). The highest proportion was roughly four times larger than the lowest proportion.

**Table 2.** Proportion of uncoded diagnoses according to the type of health insurance claim and major disease categories in ICD-10

Major disease categories		Types of HIC								

		Outpatient			Inpatient			DPC/PDPS		
		
		Uncoded	(%)	Total	Uncoded	(%)	Total	Uncoded	(%)	Total
1	Certain infectious and parasitic diseases	6858	8.5%	80 814	1078	9.9%	10 857	316	36.3%	871
2	Neoplasms	9855	11.8%	83 312	963	13.1%	7333	2767	77.9%	3550
3	Diseases of the blood and blood-forming organs and certain disorders involving the immune mechanism	1740	4.1%	42 426	432	6.5%	6675	259	38.6%	671
4	Endocrine, nutritional, and metabolic diseases	15 358	4.7%	324 783	2057	7.9%	25 951	670	34.4%	1948
5	Mental and behavioral disorders	13 890	16.1%	86 436	3016	14.4%	20 913	100	24.9%	401
6	Diseases of the nervous system	12 626	6.7%	189 406	2210	8.2%	26 942	352	30.4%	1156
7	Diseases of the eye and adnexa	38 771	13.2%	293 180	793	9.5%	8366	504	56.8%	887
8	Diseases of the ear and mastoid process	4751	15.1%	31 507	135	17.9%	754	50	55.6%	90
9	Diseases of the circulatory system	36 082	6.8%	529 060	4019	7.8%	51 322	1766	39.2%	4510
10	Diseases of the respiratory system	8811	4.2%	210 052	1363	6.7%	20 328	719	43.6%	1649
11	Diseases of the digestive system	33 659	6.7%	502 462	4174	8.2%	50 901	1114	31.7%	3517
12	Diseases of the skin and subcutaneous tissue	18 854	14.8%	127 773	2275	16.7%	13 618	212	33.7%	629
13	Diseases of the musculoskeletal system and connective tissue	42 008	8.5%	495 441	2780	10.1%	27 518	899	41.6%	2162
14	Diseases of the genitourinary system	13 156	10.6%	123 731	1370	9.8%	13 962	358	34.7%	1032
15	Pregnancy, childbirth, and the puerperium	156	11.5%	1353	21	3.1%	667	46	70.8%	65
16	Certain conditions originating in the perinatal period	34	8.3%	408	7	2.2%	312	24	72.7%	33
17	Congenital malformations, deformations, and chromosomal abnormalities	3155	19.5%	16 216	154	14.4%	1071	35	47.3%	74
18	Symptoms, signs, and abnormal clinical and laboratory findings not elsewhere classified	14 075	11.0%	127 868	2057	10.2%	20 109	366	26.9%	1361
19	Injury, poisoning, and certain other consequences of external causes	18 358	17.8%	102 924	2886	19.7%	14 666	1484	62.5%	2373

DPC/PDPS, Diagnosis Procedure Combination/Per-Diem Payment System; HIC, health insurance claim; ICD-10, the International Statistical Classification of Diseases and Related Health Problems, 10th Revision.

The number of uncoded diagnoses with text documentation that were unable to be classified according to the disease categories in the ICD-10 was 27 725. Major disease category 20 (External causes of morbidity and mortality) was not found in outpatient or inpatient HICs. There were 58 125 diagnoses in the DPC/PDPS HICs, but no diagnoses were uncoded diagnoses in this disease category.

For outpatient medical care HICs, *Diseases of the blood and blood-forming organs and certain disorders involving the immune mechanism* (Major disease category 3) had the lowest proportion of uncoded diagnoses (4.1%). For inpatient medical care, *Certain conditions originating in the perinatal period* (Major disease category 17) had the lowest proportion of uncoded diagnoses (2.2%). For DPC/PDPS, *Mental and behavioral disorders* (Major disease category 5) had the lowest proportion of uncoded diagnoses (24.9%). For all three types of HICs, the maximum value was roughly three times as large as the minimum.

The proportion of uncoded diagnoses varied according to disease category. The proportion of uncoded diagnoses for *Injury, poisoning, and certain other consequences of external causes* was the second and fourth largest among outpatient and DPC/PDPS HICs, respectively. The proportion of uncoded diagnoses for *Diseases of the ear and mastoid process* (Major disease category 8) was the fourth, second, and sixth largest in outpatient, inpatient, and DPC/PDPS HICs, respectively. The proportion of uncoded diagnoses for *Diseases of the blood and blood-forming organs and certain disorders involving the immune mechanism* (Major disease category 3) was the third smallest in outpatient HICs.

## DISCUSSION

The present study was the first in Japan to investigate the proportion of uncoded diagnoses in computerized health insurance claims according to disease categories. There are two major findings: the proportion of uncoded diagnoses differed by the type of HIC, and the proportion of uncoded diagnoses differed by disease categories.

The proportion of uncoded diagnoses was lowest in outpatient medical care HCIs and highest in DPC/PDPS HCIs. This might be explained by differences in incidence and prevalence of specific diseases in hospitalized and non-hospitalized patients, and the different characteristics between the patients hospitalized in medical facilities providing ordinary inpatient care and those in medical facilities using the DPC/PDPS. DPC/PDPS was introduced only in hospitals certified for acute inpatient care. Labor costs for diagnosis coding are higher for the medical facilities using DPC/PDPS because these facilities handle more complicated and rarer diseases, such as *Congenital malformations, deformations, and chromosomal abnormalities*.

Some medical facilities may lack the motivation to complete coding because there is no penalty for submitting uncoded diagnoses. This may be especially true for the medical facilities certified for DPC/PDPS because the hospitalization charges per day are determined according to the principal diagnoses. Therefore, medical facility staff may lack the motivation to code all but the diagnoses that are the most expensive to treat.

The diagnoses on HICs are based on information described in medical records. Therefore, some diagnoses, especially *Diseases of the ear and mastoid process* and *Injury, poisoning, and certain other consequences of external causes*, might include different information about the disease sites, such as the right side or left side of the body. However, the diagnosis codes for Japanese computerized HICs do not distinguish such sites because they are based on the ICD-10. Thus, diagnoses with accompanying information on site might be difficult to code. This point is supported by the finding that the proportion of uncoded diagnoses was relatively low in diagnoses with little or no need to distinguish the sites on the body, such as *Diseases of the blood and blood-forming organs and certain disorders involving the immune mechanism*. Detailed investigation of the role of easy-to-code diagnoses on the uncoded HICs in Japan is required.

The proportion of uncoded diagnoses was 9.6%. This proportion is slightly larger than that found in our previous report, which analyzed HICs submitted to the NHIO of Kumamoto Prefecture in December 2010.^16^ The proportion of uncoded diagnoses was expected to decline as medical facilities became more accustomed to coding diagnoses under the mandatory electronic HIC submission requirement. Given our results, the proportion of uncoded diagnoses should be analyzed periodically to assess any changes.

There are some limitations in this study. First, this study did not assess the validity of diagnoses described in the HICs^17^^,^^18^ or accuracy of coding for the diagnoses,^19^ and the validity of this study rests on the validity of coded diagnoses. The cited studies, which were not conducted in Japan, used medical charts^17^^,^^19^ and telephone interviews^18^ to obtain the necessary data. In Japan, HICs contain not only confirmed diagnoses but also unconfirmed or disproved diagnoses, because each clinical procedure must be justified by a corresponding diagnosis; as a result, rule-out diagnoses are included in the HICs to ensure reimbursement for these clinical procedures, even when the results show that the suspected diseases are not present.^20^^,^^21^ Assessment of the validity of diagnoses described on HICs and accuracy of coding for the diagnoses on HICs in Japan awaits further investigation.

Second, the analysis was conducted only with HICs for patients covered under the National Health Insurance and the medical system for the elderly aged 75 years and older. Therefore, the data from the HICs analyzed in this study are not necessarily representative of the total population. However, we can assume that the current results are generalizable because the format of HICs in Japan is uniform regardless of the patients’ insurance.

Third, I analyzed HICs submitted for services provided only in May. This is consistent with other health statistics studies that use HIC data in Japan, such as the Social Insurance Claims Survey and the National Health Insurance Medical Benefit Survey, but it assumes that seasonal fluctuations are at a minimum. After the mandatory submission of electronic HICs, it has become easier to analyze an entire year of HICs. Further investigation of the proportion of uncoded diagnoses in electronic HICs over a longer period is needed.

In conclusion, the proportion of uncoded diagnoses in computerized health insurance claims in Japan accounts for 9.6% of the total diagnoses. Further, the proportion of uncoded diagnoses differed by the type of HIC and the disease category. These findings indicate that excluding uncoded diagnoses may introduce bias into Japanese health statistics that are based on computerized HICs. The causes behind the presence of uncoded diagnoses in computerized health insurance claims should be identified to improve Japanese health statistics based on computerized HIC data.

## ONLINE ONLY MATERIAL

Abstract in Japanese.
