# Supplementary material for: The Proportion of Uncoded Diagnoses in Computerized Health Insurance Claims in Japan in May 2010 According to ICD-10 Disease Categories
Source: J Epidemiol. 2014 Sep 5;24(5):392–6. doi: 10.2188/jea.JE20130194 (PMC4150010; doi:10.2188/jea.JE20130194)
Supplement: Abstract in Japanese. [file je-24-392-s001.pdf]

電子化された診療報酬明細書（2010 年 5 月診療分）における疾病大分類別の未コード化傷病名割合に関する分析

谷原真一（福岡大学医学部衛生公衆衛生学）

【はじめに】診療報酬明細書（レセプト）の電子化が進展したが未コード化傷病名はレセプトを用いた政府統計や調査における活用が困難である。未コード化傷病名の存在がレセプト分析に与える影響を検証する目的で国際疾病分類第 10 版（ICD10）の大分類別に未コード化傷病名割合を検討した。
【方法】熊本県国民健康保険団体連合会にオンラインもしくは電子媒体で提出された 2010 年 5 月診療分のレセプト（医科入院外、医科入院、DPC/PDPS）に記載された全ての傷病名を ICD10 対応標準病名マスターに沿ったコード化傷病名と未コード化傷病名に分類した。コード化傷病名は ICD10 の大分類に沿って再分類した。未コード化傷病名は当該傷病名に対応してテキスト入力された傷病名を用いて分類した。医科入院外、医科入院、ＤＰＣの種類別に大分類別の未コード化傷病名の割合を集計した。
【結果】分析に用いた傷病名総数 3,804,246 件の内 363,753 件 (9.6%)が未コード化傷病名であった。医科入院外、医科入院、DPC/PDPS の未コード化傷病名割合はそれぞれ 9.3%、10.9%、 14.2%とレセプトの種類によって未コード化傷病名割合が異なっていた。大分類別では最大値「先天奇形、変形及び染色体異常」（大分類 20）の 19.3%と最小値「呼吸器系の疾患」（大分類 10）の 4.7%に 4 倍以上の格差を認めた。
【結論】未コード化傷病名の出現頻度はレセプトの種類や傷病大分類によって異なっていた。電子化されたレセプトを分析する上で未コード化傷病名の存在は無視できないと考えられた。

キーワード：診療報酬明細書、未コード化傷病名、ICD10
